# Correlations between Motor Symptoms across Different Motor Tasks, Quantified *via* Random Forest Feature Classification in Parkinson’s Disease

**DOI:** 10.3389/fneur.2017.00607

**Published:** 2017-11-14

**Authors:** Andreas Kuhner, Tobias Schubert, Massimo Cenciarini, Isabella Katharina Wiesmeier, Volker Arnd Coenen, Wolfram Burgard, Cornelius Weiller, Christoph Maurer

**Affiliations:** ^1^Department of Computer Science, University of Freiburg, Freiburg, Germany; ^2^BrainLinks BrainTools, Cluster of Excellence, University of Freiburg, Freiburg, Germany; ^3^Department of Neurology and Neuroscience, Medical Center, University of Freiburg, Freiburg, Germany; ^4^Medical Faculty, University of Freiburg, Freiburg, Germany; ^5^Department of Stereotactic and Functional Neurosurgery, Medical Center, University of Freiburg, Freiburg, Germany

**Keywords:** Parkinson’s disease, motion, deep brain stimulation, random forest, sensor suit

## Abstract

**Background:**

Objective assessments of Parkinson’s disease (PD) patients’ motor state using motion capture techniques are still rarely used in clinical practice, even though they may improve clinical management. One major obstacle relates to the large dimensionality of motor abnormalities in PD. We aimed to extract global motor performance measures covering different everyday motor tasks, as a function of a clinical intervention, i.e., deep brain stimulation (DBS) of the subthalamic nucleus.

**Methods:**

We followed a data-driven, machine-learning approach and propose performance measures that employ Random Forests with probability distributions. We applied this method to 14 PD patients with DBS switched-off or -on, and 26 healthy control subjects performing the Timed Up and Go Test (TUG), the Functional Reach Test (FRT), a hand coordination task, walking 10-m straight, and a 90° curve.

**Results:**

For each motor task, a Random Forest identified a specific set of metrics that optimally separated PD off DBS from healthy subjects. We noted the highest accuracy (94.6%) for standing up. This corresponded to a sensitivity of 91.5% to detect a PD patient off DBS, and a specificity of 97.2% representing the rate of correctly identified healthy subjects. We then calculated performance measures based on these sets of metrics and applied those results to characterize symptom severity in different motor tasks. Task-specific symptom severity measures correlated significantly with each other and with the Unified Parkinson’s Disease Rating Scale (UPDRS, part III, correlation of *r*^2^ = 0.79). Agreement rates between different measures ranged from 79.8 to 89.3%.

**Conclusion:**

The close correlation of PD patients’ various motor abnormalities quantified by different, task-specific severity measures suggests that these abnormalities are only facets of the underlying one-dimensional severity of motor deficits. The identification and characterization of this underlying motor deficit may help to optimize therapeutic interventions, e.g., to “automatically” adapt DBS settings in PD patients.

## Introduction

The most frequently used tool for clinically assessing Parkinson’s disease (PD) is the revised version of the Unified Parkinson’s Disease Rating Scale [International Parkinson and Movement Disorder Society (MDS)-UPDRS, 1]. The Unified Parkinson’s Disease Rating Scale (UPDRS) combines numerous clinical scales and questionnaires regularly used in clinical routine and in studies to evaluate the presence, severity, and progression of PD symptoms (1). However, the disadvantages of the UPDRS are that it is very time-consuming, its inter- and intra-rater variability, dependence on location, limitations involving regularly repeating the assessments, and the lack of quantitative outcomes (2). Adjusting deep brain stimulation (DBS) parameters in PD patients still relies on subjective estimations of patients’ clinical state, despite the vast motor symptoms and adjustable parameters.

Motion capture techniques were recently introduced to characterize motor behavior in PD more objectively (2–4). Early attempts focused on evaluating individual symptoms (5, 6), using either single (7–9) or multiple-sensor systems (6, 10–13). Overall, assessing motor symptoms using motion capture techniques seemed to correlate well with specific clinical tests for motor symptoms, such as tremor, bradykinesia, or dyskinesia (14). Since these assessments are usually closely related to the specific clinical tests, less is known about how these test results relate to more everyday life motor activities, such as walking, bending over, goal-directed hand movements, etc. Moreover, we do not yet know how these symptoms correlate with each other in the individual patient.

One of the benefits of motion capture techniques is that machine-learning approaches can be implemented to handle the growing amount of data (15). Some authors applied machine-learning approaches to automatically differentiate PD patients from healthy controls or to classify levels of motor impairment using various methods and parameters [(16, 17): gait parameters, (18): quantitative EEG, (19): computer vision techniques]. Recently, Bernad-Elazari et al. (20) examined the benefit of a single body-fixed sensor to discriminate between PD patients and healthy controls. Machine-learning approaches have also been investigated to monitor tremor, dyskinesia, or bradykinesia in PD patients (21, 22). However, we are unaware of any study taking machine-learning approaches to derive a continuous measure of motor performance across many different motor tasks.

Earlier studies already aimed to evaluate motor abnormalities that directly target certain motor tasks, e.g., free stance, as the accurate identification of balance abnormalities is of high clinical relevance in PD (23, 24). Even a seemingly simple task like the real-world detection of PD patients’ falls is technically challenging. Numerous algorithms, devices, and device locations [chest, waist, shin, or wrist (25, 26)] have been tested to improve the accuracy of fall detection in PD patients. Still, these studies reveal significant variability in the outcomes they measured.

Objective assessments of PD patients’ gait abnormalities may help the clinician to evaluate the efficacy of therapeutic interventions and are potentially useful as biological markers for PD’s diagnosis, prognosis, and progression (27–29). The validities of various algorithms that detect discrete gait characteristics in the laboratory have already been investigated (14, 30–34). However, how these measures relate to each other and to other motor abnormalities in PD patients remains open to debate.

More general clinical tests of functional mobility such as the Timed Up and Go Test [TUG; (35–37)] combine movements that invariably incorporate postural transitions. Mean turn velocity, slower walking and turning, shorter steps, and lower cadence distinguished PD from controls (35, 37) and also revealed greater sensitivity to dysfunction than most other clinical rating scores (35, 38). Again, it remains unclear how closely the Timed Up and Go elements correlate with each other and with other motor abnormalities in the individual PD patient.

In this study, we aimed to detect abnormal motor behavior of PD patients performing several different motor tasks in two clinical conditions (DBS switched-off, -on). We extracted metric values from motion capture suit data and demonstrated that each performed task is best characterized by a specific metric. Then, we combined all metrics with a Random Forest approach and calculated the most discriminative markers for abnormality in each motor task. These Random Forests, enhanced by probability distributions, were then used as symptom severity measures. Finally, we evaluated how well these task-specific severity measures correlated with each other.

## Materials and Methods

### Subjects and Data

**Figure 1:**
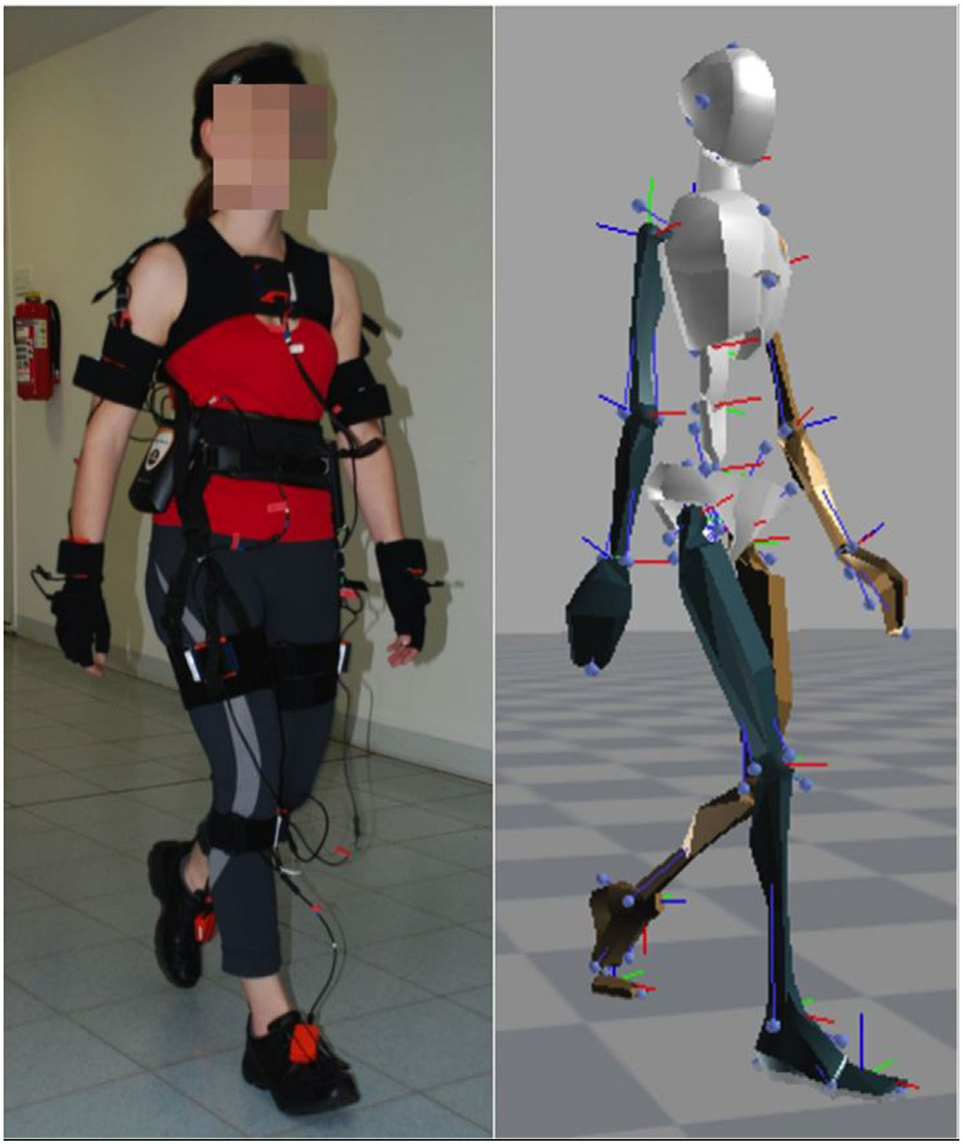
XSens motion capture suit and corresponding avatar.

We used the XSens motion capture suit to record the motion data of 26 healthy subjects [11 females, 15 males, mean age 54.7 ± 8.8 years (±SD)] and 14 PD patients (5 females, 9 males, mean age 59.1 ± 11.1 years, for detailed information, see Table 1). The motion capture suit consists of 17 MEMS (microelectromechanical systems) with outputs based on the fusion of signals stemming from 3D inertial measurement units (IMUs), ie, linear accelerometers, 3D magnetometers, and 3D rate gyroscopes. This system’s raw outcome is velocity traces. It records data with a frame rate of 120 Hz and stores positional and rotational data of 23 body parts (Figure 1).

**Table 1:** Data of patients with Parkinson’s disease.

Patient	Gender	Age (years)	Disease duration (years)	Time since Stim. Impl. (months)	LEDD (mg)	UPDRS motor score	
						DBS off	DBS on
1	F	74	11	1	0	20	15
2	F	69	8	1	0	29	20
3	M	74	12	1	607	43	22
4	F	49	9	1	475	51	32
5	F	53	11	1.5	700	38	29
6	M	57	14	1	349.5	47	15
7	M	40	10	1	475	61	16
8	M	43	10	2	1,308.3	28	15
9	M	66	21	1	717.5	42	35
10	M	55	25	1	0	43	36
11	F	61	14	1.5	525.75	34	12
12	M	72	3	1.5	0	36	20
13	M	52	14	2	0	21	22
14	M	62	26	1.5	379.25	36	16
Mean		59.1	13.4	1.3	395.5	37.8	21.8
SD		11.1	6.4	0.4	367.30	11.3	8.0

*F, female, M, male, Stim. Impl., stimulator implantation, UPDRS, Unified Parkinson’s Disease Rating Scale, DBS, deep brain stimulation, LEDD, levodopa-equivalent doses*.

Parkinson’s disease patients were recruited during the initial programming for bilateral DBS of the subthalamic nucleus. Stimulator implantation followed established guidelines. The study protocol involved the Timed Up and Go Test (TUG), the Functional Reach Test (FRT), a 10-m Walk Test (39), a 90° turn to right and to the left, and a hand coordination task (pouring water from a glass in the right hand to a glass in the left hand and *vice versa*). The TUG and the FRT were repeated three times. Our analysis was based on 20 to 30 steps during straight walking, two 90° turns for each direction, and 12 hand coordination tasks. PD patients were recorded in both the stimulation switched-off (off DBS) and switched-on (on DBS) condition while taking their regular l-Dopa medication. The levodopa-equivalent doses were calculated according to Ref. (40).

In addition, patients were assessed with the Unified Parkinson’s Disease Rating Scale [International Parkinson and MDS-(part III), 1]. The UPDRS motor exam (part III) represents the patients’ state by evaluating multiple motions like walking and hand coordination. Additionally, the UPDRS (part III) rates the stiffness of limbs or the ability to regularly perform repeated movements.

All subjects and patients gave their written informed consent and their data were included anonymized according to the Declaration of Helsinki. We carried out this study in accordance with the guidelines of the Ethics Committee of the Medical Center, University of Freiburg and national guidelines. The Ethics Committee of the Medical Center, University of Freiburg approved the study protocol.

### Metrics

To calculate our metrics, all motor tasks except FRT were segmented into simpler elements, e.g., single steps, single hand movements for pouring water, or sub-tasks of the TUG like stand-up, turn, and sit down.

For these individual elements of tasks we use velocity time series from each body segment to derive single metrics. The Joint Activity (JA, dimensionless) represents the distance between an individual’s motion velocity pattern and the average patterns of healthy subjects. It covers all metrics based on velocity traces and yields the degree of abnormality in terms of deviation from the normal range. This deviation represents a single segment velocity trace’s different timing and movement profile during a sub-task. The distance is the probability that a given velocity pattern will match the average motion pattern of healthy subjects (41). As a single velocity trace could permanently remain within the range of normal behavior and still be highly abnormal due to, for example, high-frequency oscillations, we extended the metric “Joint Activity” by a set of smoothness- and frequency-related metrics. Our metrics describing the trajectory’s smoothness included the normalized mean absolute jerk [NMAJ (1/s^2^)], dimensionless jerk [DJ (dimensionless)], log dimensionless jerk [LDJ (dimensionless)], root mean square jerk [RMSJ (m/s^3^)], and speed arc length [SpAL (dimensionless) (42)]. For details concerning the calculation of these metrics, see Supplementary Material. A metric representing the frequency content of a movement was spectral arc length [SAL (dimensionless)].

We apply the set of metrics to each of an individual’s various body parts. Here, a sample consists of the 23 body part velocity profiles from a subject in one of the sub-tasks, e.g., a single step. Overall, each sample yields a 161 dimensional metric vector, in other words 7 metrics for 23 body parts.

### Random Forests with Probability Distributions

Due to the high-dimensional data vectors, it is difficult to identify the most useful features for characterizing pathological movements. To overcome this challenge, we compute for each sub-task a Random Forest (43) which combines the aforementioned metric vectors and filters the highly dimensional data to compute classification and performance measures. Our Random Forest approach consists of multiple decision trees. In the learning phase we optimize each decision tree on a subset of the training samples with a subset of metrics, i.e., we use 40% randomly chosen samples, i.e., task elements, from the training set and 11 randomly chosen metrics for each tree. Each decision tree is composed of nodes in which we split the training data into two subsets. Each node separates the training data through a randomly chosen threshold on a single metric. From all the decision tree’s metrics, we choose the one that provides the most information. We repeat this procedure from node to node as long as we have a non-unique data set or reach a certain depth.

The Random Forest calculates the class of a new sample by supplying each decision tree with the sample data to obtain a single outcome. Furthermore, the Random Forest applies a majority vote over all decision trees to represent the final result. This leads to a binary decision.

We trained the algorithms to create a contrast between healthy and maximum pathological state, e.g., PD off DBS. The purpose of this binary decision was to apply it as a quality measure to compare different metrics and combinations of metrics derived from Random Forests on the basis of their accuracy, that is, their recognition rate to identify the correct subject group (see Table 2).

**Table 2:** Accuracies for each executed task over all metrics.

	JA	DJ	LDJ	NMAJ	RMSJ	SAL	SpAL	Combined
Steps	**85.7**	59.6	60.1	57.0	80.8	**88.7**	62.4	**89.2**
Curve	80.0	91.0	**92.0**	67.0	79.0	77.0	**94.0**	**92.0**
Functional reach	66.0	73.0	73.4	**86.9**	72.6	75.2	**77.8**	**86.0**
Hand task	69.2	82.6	82.4	75.2	74.5	**83.1**	**84.6**	**87.5**
Stand-up	77.8	79.1	77.1	66.4	**87.9**	69.1	**86.5**	**94.6**
Turn	75.1	74.4	77.1	63.7	**83.8**	71.1	**84.5**	**89.9**
Sit down	**87.9**	81.8	83.2	82.5	79.8	67.7	**84.5**	**88.5**

*The best metrics for each task are highlighted. Accuracies refer to Parkinson’s disease patients with deep brain stimulation switched-off compared to healthy control subjects. The values result from 7 + 1 cross-validation tests*.

*JA, joint activity (dimensionless); DJ, dimensionless jerk; LDJ, Log dimensionless jerk; NMAJ, normalized mean absolute jerk (1/s^2^); RMSJ, root mean square jerk (m/s^3^); SAL, spectral arc length (1/s^3^), SpAL, speed arc length (dimensionless)*.

### Symptom Severity Measure

We then go one step further and compute continuous performance measures in the interval [0, 1]. In so doing, we extend each node *via* a probability distribution over the nodes’ specific subset of training data and, while supplying the nodes of the decision tree with a new sample, multiply the sample’s probabilities in the node’s particular distribution. We obtain a sample data point’s performance measure by summing up each decision tree’s probabilities and normalizing the result. The purpose of this performance measure was to measure symptom severity.

More specifically, the Random Forests’ training was based on a data set that represents both extremes of a disease: healthy subjects and PD patients off DBS. This provides us with a contrast to create a measure that describes all states in between. The Random Forest itself “learns” an exclusively either-or classification, while our performance measure utilizes the underlying tree structure to calculate a real-valued number between 0 and 1. Furthermore, the composition of decision trees that are trained on a reduced training set size and with a subset of metric values enhances the generalization capabilities and prevents overfitting. We also carry out a leave-one-subject-out cross-validation. Hence, we remove one subject from the training set, teach our Random Forest on the training set and test its performance on the missing data sample. This procedure is repeated for each subject, and the resulting values depict our algorithm’s performance.

One of the upsides of a Random Forest is its ability to detect high-level correlations between different metrics. Through the nested transitioning over many layers in a tree, one obtains a multidimensional threshold function that sets metrics in relation to each other. Hereby, PD motor abnormalities like asymmetric motor behavior are automatically detected and used if they carry additional information.

### Correlation Analysis

We compared our results of combined metrics with the UPDRS (part III) in order to verify their reliability. The correlation of performance measures with each other and the UPDRS (part III) were analyzed *via* the Pearson correlation test.

## Results

### Classification Accuracy for Different Tasks

Table 2 illustrates the results of all 7 + 1 cross-validation tests of several tasks with different metrics and the combination of all metrics in one Random Forest between patients with off DBS and healthy controls. The seven tasks and sub-tasks are characterized by different sets of metrics indicating diverse motor abnormalities. In general, SpAL yielded high accuracy for all tasks except walking (steps). Specifically, walking a 90° curve was best characterized by SpAL (94%) and Log Dimensionless Jerk (LDJ, 92%), whereas turning was best characterized by SpAL (84.5%) and RMSJ (83.8%). SAL and JA displayed the highest accuracy during walking (steps, 88.7 and 85.7%, respectively). The highest accuracy regarding the FRT was achieved by SpAL (77.8%) and NMAJ (86.9%), whereas the Hand Coordination Task’s highest accuracy was achieved by SpAL (84.6%) and SAL (83.1%). Standing up and sitting down were best characterized by SpAL (Stand-up: 86.5%, sit down 84.5%), RMSJ (Stand-up, 87.9%), and JA, respectively (Sit down, 87.9%). The agreement rate between different task-specific measures ranged from 79.8 to 89.3% with the highest agreement rate between standing up and the 10-m Walk Test.

We computed combined measures for each task using the Random Forest. The combined measures revealed similarly high accuracy over all tasks and sub-tasks. The highest combined accuracy (94.6%) was achieved for the task “standing up.” This corresponded to a sensitivity of 91.5% to detect a PD patient off DBS, and a specificity of 97.2% representing the rate of correctly identified healthy subjects.

### Symptom Severity Measures

**Figure 2:**
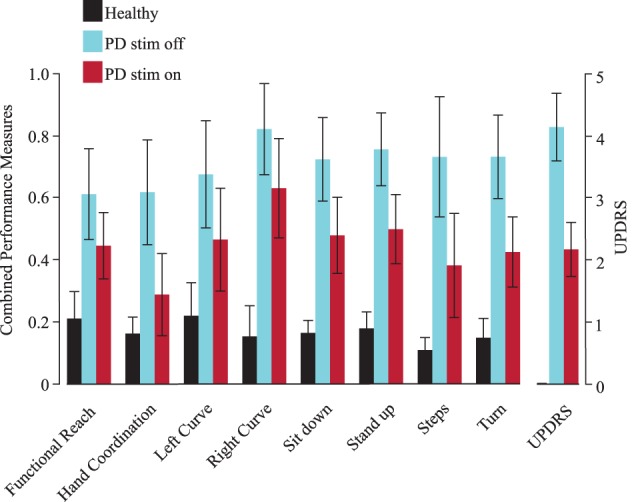
Combined performance measures of all subjects for each task. The Unified Parkinson’s Disease Rating Scale [Unified Parkinson’s Disease Rating Scale (UPDRS), part III, right] is shown as a verifiability measure. Black represents healthy subjects, blue patients with deep brain stimulation (DBS) switched-off and red with DBS switched on. Error bars refer to the SE. PD, Parkinson’s disease (patients), stim off, DBS switched-off, stim on, DBS switched on.

Using the decision tree’s probabilities, we calculated performance measures that represent the severity of symptoms based on the average of likelihoods from each decision tree to be a PD patient off DBS. When applying the performance measure on PD patients on DBS, we observed an average value of 53.3%—halfway between healthy and PD off DBS, as expected. Comparison of these combined performance measures with the UPDRS (part III) showed that their outcomes are similar (Figure 2). In all combined performance measures as well as in the UPDRS (part III), healthy control subjects scored low, followed by PD patients on DBS. PD patients off DBS got the highest scores.

### Improvement through DBS

**Figure 3:**
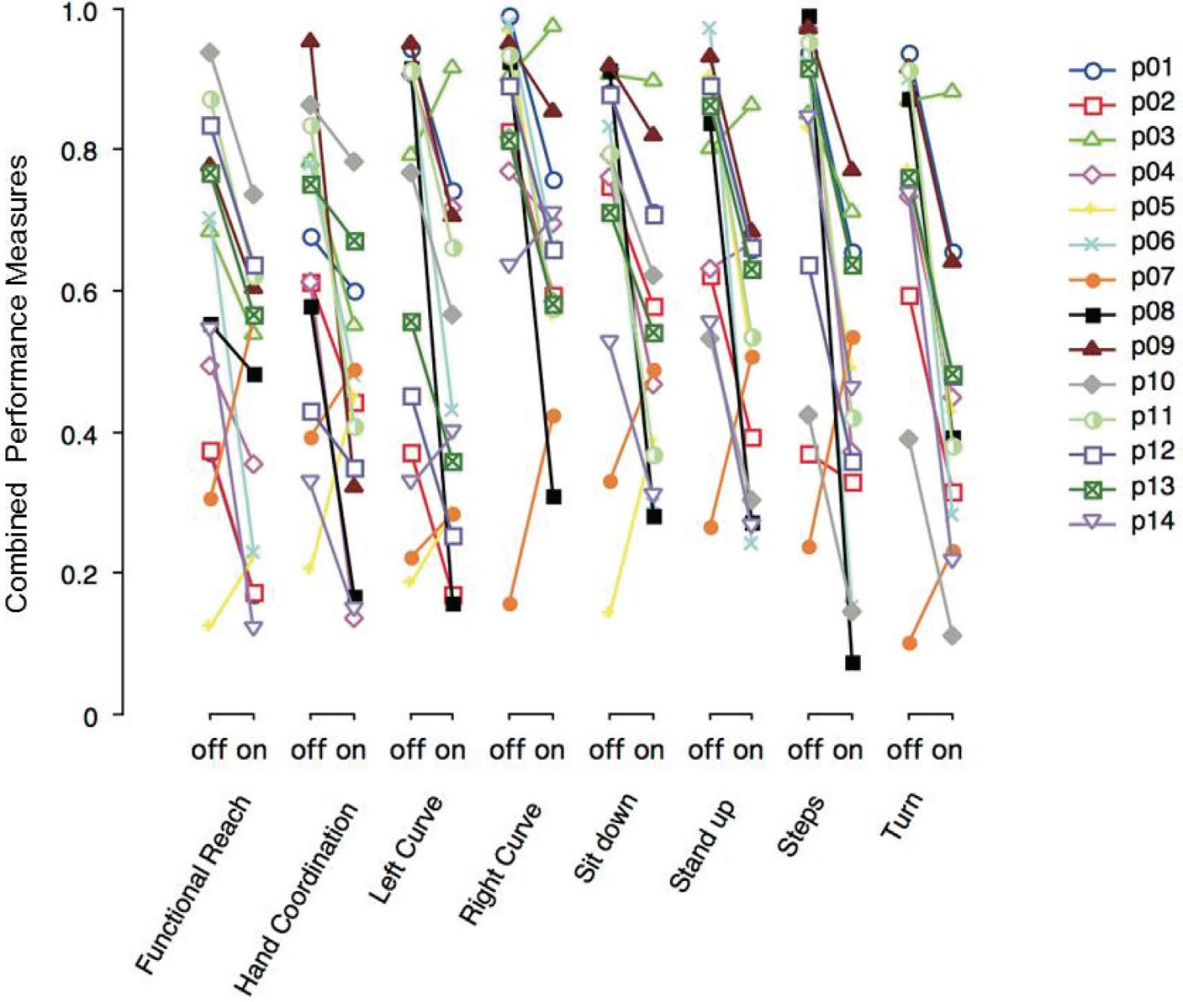
Combined performance measures of Parkinson’s disease (PD) patients with deep brain stimulation (DBS) switched-off or -on, separated by task. Each PD patient (p01–p14) is represented by a particular symbol and color.

Figure 3 shows the combined performance measures of various tasks performed with DBS switched-off or on. Each PD patient (p01–p14) is depicted with a particular symbol and color. Most PD patients show lower performance measure values on DBS across all tasks. Note that the severity of motor symptoms and the effect of DBS in a single patient tended to be similar across different tasks.

### Correlation Analysis

Correlating single metrics across different motor tasks leads to certain groups of motor tasks that correlate closely with each other. For example, the JA metrics derived from certain motor tasks (stand-up, turn, sit down, curve, and steps) correlated closely, while JA metrics derived from the hand coordination task or from functional reach did not correlate significantly. No single metric correlated significantly across all motor tasks.

**Figure 4:**
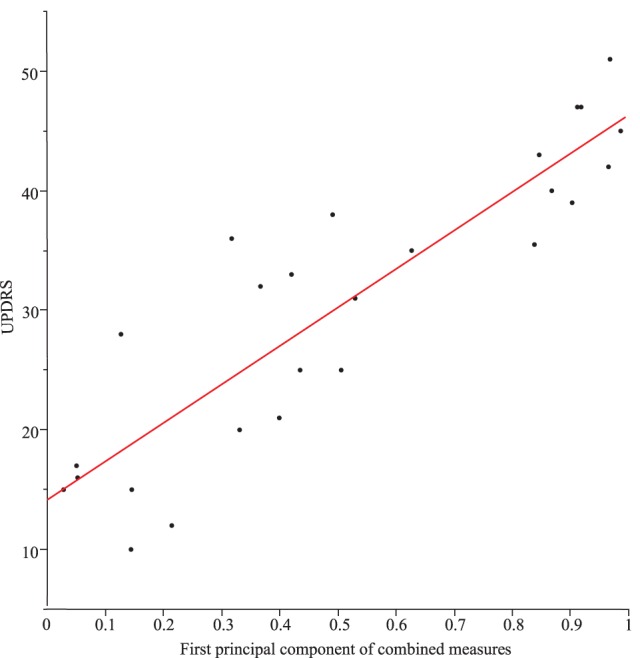
Correlation between Unified Parkinson’s Disease Rating Scale (UPDRS) (part III) and the first principal component of the combined measures from the different tasks (*r*^2^ = 0.79) for Parkinson’s disease patients with DBS switched-off and switched-on.

However, if we take from each motor task the single best metric (apart from the combined, see Table 2), all different, task-specific metrics correlated significantly with each other (all *p* values < 0.004). When correlating individual combinations of metrics (symptom severity measures) from each motor task, the correlations across all motor tasks were even higher (*p* values < 0.0003). Moreover, task-specific combinations of severity measures correlated significantly with the UPDRS (part III). The first principal component alone represents 72% of the total variance of the data field and correlated significantly with the UPDRS (part III; *r*^2^ = 0.79, *p* < 0.0001, see Figure 4).

## Discussion

In this investigation, we took a data-driven approach aiming to identify symptom severity measures to comprehensively characterize motor abnormalities in PD. We examined motor behavior during several different everyday motor tasks [sit-to-stand, turning, stand-to-sit (Timed Up and Go Test, TUG), walking straight, walking a 90°curve, leaning forward as much as possible (FRT), and a hand coordination task]. We selected these motor tasks specifically to represent the most relevant impairments in PD patients’ quality of life (2, 14).

Data were recorded with a motion capture suit comprising 17 MEMS (microelectromechanical systems) with outputs based on the fusion of signals stemming from IMUs, magnetometers, and gyroscopes. Velocity traces were the raw outcome of this system. We used the data of all 17 MEMS for further analysis, as we did not want to bias the purely data-driven approach by choosing or excluding any segment arbitrarily. The Xsens sensor suit used here consisted of straps rather than a “suit,” was “light weighted,” and did not hamper any movement.

The minimum number of sensors required to capture reliable whole-body motion data are still unknown. Most investigators have applied few sensors to characterize single body parts [e.g., Ref. (44)]. Semwal et al. (45) used eight sensors to capture full-body movements, but enriched sensor data with information on natural body posture. Future studies are needed to clarify this issue.

One limitation of this study is our relatively low number of healthy subjects (*n* = 26) and PD patients (*n* = 14) that could restrict our findings’ generalizability. However, our approach was designed to identify severity measures of different motor symptoms in different motor tasks in individual PD patients. Moreover, the findings reported herein are based on a substantial amount of data, i.e., 23 body-part velocity profiles for a single element in a task (e.g., one step) and a 161 dimensional metric vector. Another limitation is that our metrics are based on segment velocity traces and we consequently miss the information from the initial positional offset in all joints. However, all other displacement-related abnormalities should be captured by these joint velocity traces.

Since PD patients’ intended movement amplitudes or velocities are not known prior to the movement, we came up with a new metric named JA, which is based on the likelihood of a single joint velocity trace falling outside the normal range (41). JA may be abnormal even when the data distribution in healthy subjects is more complex, such as when it is bimodal. Theoretically speaking, a behavior can be considered abnormal if it is halfway between two normal ranges. In that respect, we believe that JA is a more broadly applicable measure for abnormality than too small or too large movement amplitudes, velocities, or ranges with respect to the normal subjects. Using many velocity traces from different joints, JA-related findings could also cover abnormal multisegmental control strategies in comparison to healthy subjects (46).

As a single velocity trace could remain within the range of normal behavior and still be abnormal due to high-frequency oscillations, we included additional motor-performance metrics associated with smoothness (e.g., NMAJ) or frequency content (i.e., Spectral Arc Length, SAL) of movement trajectories. Smoothness measures are known to be specifically abnormal in PD patients (14). Moreover, due to diverse calculation properties and normalization procedures, the smoothness measures used here characterize different traits of motor behavior.

Concerning the identification of a single metric that optimally distinguishes between normal and abnormal behavior, JA proved to be most successful in characterizing those motor tasks that involved whole-body movements (stand-up, turn, sit down, curve, and steps). In contrast, functional reach and hand coordination were characterized less precisely. On the other hand, there is evidence that the metric “Spectral Arc Length” covers abnormal arm movements in stroke patients (42). Here, SAL delivered high precision in PD patients’ walking. However, other metrics outperformed it in all the other motor tasks. Interestingly, we found that “Speed Arc Length” was the most precise metric for many motor tasks (walking a 90° curve, 180° turning, hand coordination) while it was less precise for walking straight and functional reach. “Normalized Mean Absolute Jerk” outperformed other metrics for functional reach. The metric with large content of velocity and displacement, namely “Root Mean Square Jerk,” revealed high precision for standing up and turning.

With the help of Random Forests, we sought optimal combinations of metrics to correctly classify PD patients off DBS and healthy subjects for each motor task separately. We used the accuracy of these metrics to compare the classification power of different metrics. Accuracy values were calculated using a leave-one-subject-out cross-validation. This procedure was repeated for each subject, and the values were averaged, resulting in a given Random Forest’s average accuracy. In general, we observed that motor abnormalities in PD patients off DBS can be very precisely characterized *via* a set of metrics. The highest accuracy (94.6%) was achieved for standing up. This corresponded to a sensitivity of 91.5% to detect a PD patient off DBS, and a specificity of 97.2% representing the rate of correctly identified healthy subjects. Thus, when testing the metrics to identify the one that optimally characterizes pathological behavior, we detected differences between tasks.

We then used the new metrics generated by Random Forests, which were derived from the classification results, to generate performance measures based on the mean of many recognition decisions between “zero” and “one” across the 161 metrics. They are related to the sum of probability distributions over the nodes of the decision trees. The purpose of this performance measure was to generate a measure for symptom severity. When applying the performance measure on PD patients on DBS, we identified a symptom severity of 53.3%, as expected.

Since the optimal metric for measuring symptom severity in each motor task is different, one single new metric does not lead to high correlations across tasks. The low correlations between single metrics could have two potential sources. First, PD patients might exhibit individual combinations of deficits. Second, one single measure may be unsuitable for every motor task. To resolve this problem, we compared correlations between single metrics across tasks with correlations of task-specific Random Forests. The fact that the task-specific Random Forests correlated significantly better than any single measure indicates that the severities of PD patients’ individual motor abnormalities in different motor tasks are highly interdependent. In other words, PD patients do not reveal a broadly scattered symptom severity across different tasks. This includes balance, fine motor skills, trunk movements, walking, and turning.

We suggest that PD patients’ severity of motor symptoms is one-dimensional, i.e., they suffer from a common underlying motor deficit. This underlying motor deficit is an abstract measure, related to the first principal component across all motor abnormalities. It is related, e.g., to the slowness of standing up, smoothness of a fine motor skill of the hand, step length when walking. It is not a sum across all motor abnormalities, but the severity of motor abnormality in each motor task separately. This common motor deficit seems to affect different motor tasks in different ways. Consequently, the way how these motor abnormalities are optimally assessed, differs between tasks. With the help of task-specific extended Random Forests, we are capable of successfully deriving the common motor deficit no matter which task the PD patient is currently performing.

We plan to apply this common motor deficit measure to optimize therapeutic interventions, e.g., to adjust parameters for DBS electrodes (electrode location, amplitude, frequency, pulse width, direction of field). For a more automated assessment of PD patients’ current motor state, we aim to complement the approach presented here by algorithms able to recognize the ongoing motor task in order to select the correct task-specific extended Random Forest.

## Ethics Statement

All subjects and patients gave their written informed consent and their data were included anonymized according to the Declaration of Helsinki. We carried out this study in accordance with the guidelines of the Ethics Committee of the Medical Center, University of Freiburg and national guidelines. The Ethics Committee of the Medical Center, University of Freiburg approved the study protocol.

## Author Contributions

AK and CM contributed to the concept and design of the work, analysis and interpretation of data, and drafted the manuscript. AK was also responsible for data acquisition. TS and MC contributed to conceiving and designing the work, to acquiring data, and to critically revising the manuscript. IW drafted and edited the final manuscript for submission and revised the work critically. VC, WB, and CW contributed to the concept and critical revision of the work. All authors approved the final manuscript.

## Conflict of Interest Statement

The authors declare that the research was conducted in the absence of any commercial or financial relationships that could be construed as a potential conflict of interest.
