# Supplementary material for: Correlations between Motor Symptoms across Different Motor Tasks, Quantified via Random Forest Feature Classification in Parkinson’s Disease
Source: Front Neurol. 2017 Nov 14;8:607. doi: 10.3389/fneur.2017.00607 (PMC5694559; doi:10.3389/fneur.2017.00607)
Supplement: Supplementary file 1 [file Data_Sheet_1.DOCX]

Supplementary Material

Correlations between motor symptoms across different motor tasks, quantified via Random-Forest feature classification in Parkinson’s disease

Andreas Kuhner, Tobias Schubert, Massimo Cenciarini, Isabella Katharina Wiesmeier, Volker Arnd Coenen, Wolfram Burgard, Cornelius Weiller, Christoph Maurer*

*** Correspondence:** Christoph Maurer

Department of Neurology and Neuroscience

Medical Center – University of Freiburg

Breisacherstr. 64

D-79106 Freiburg

Germany

Phone: +49 761 270 52380

Fax: +49 761 270 52300

Email: christoph.maurer@uniklinik-freiburg.de

# Supplementary Data

All formulas for additional metrics, first the Dimensionless Jerk:

$$m_{DJ}=-\frac{\left( t_{2}-t_{1} \right)^{3}}{v_{peak}^{2}}\int_{t_{1}}^{t_{2}} \left| \frac{d^{2}v}{dt^{2}} \right|dt$$

Log Dimensionless Jerk:

$$m_{LDJ}=-\ln\left( {-m}_{DJ} \right)$$

Speed Arc Length:

$$m_{SpAL}=-\ln\left( \int_{t_{1}}^{t_{2}} \sqrt{\left( \frac{1}{t_{2}-t_{1}} \right)^{2}+\left( \frac{d\hat{v}}{dt} \right)^{2}}dt \right),$$

with $\hat{v}=\frac{v(t)}{v_{peak}}$.

Root Mean Square Jerk:

$$m_{RMSJ}=-\sqrt{\frac{1}{(t_{2}-t_{1})}\int_{t_{1}}^{t_{2}} \left| \frac{d^{2}v}{dt^{2}} \right|dt}$$

Normalized Mean Absolute Jerk:

$$m_{NMAJ}=-\frac{1}{v_{peak}(t_{2}-t_{1})}\int_{t_{1}}^{t_{2}} \left| \frac{d^{2}v}{dt^{2}} \right|dt$$

Spectral Arc Length:

$$m_{SAL}=-\sum_{k=1}^{K_{c}-1} \sqrt{\left( \frac{1}{K_{c}-1} \right)^{2}+\left( \Delta\hat{V}\left[ k \right] \right)^{2}},$$

with $\hat{V}$ as Discrete Fourier Transformation and $K_{c}$ as its index.
